# Statistical shape analysis of tap roots: a methodological case study on laser scanned sugar beets

**DOI:** 10.1186/s12859-020-03654-8

**Published:** 2020-07-29

**Authors:** Behrend Heeren, Stefan Paulus, Heiner Goldbach, Heiner Kuhlmann, Anne-Katrin Mahlein, Martin Rumpf, Benedikt Wirth

**Affiliations:** 1 Institute for Numerical Simulation, University of Bonn, Endenicher Allee 60, Bonn, 53115 Germany; 2 Institute of Sugar Beet Research, Germany, Holtenser Landstr. 77, Göttingen, 37079 Germany; 3 INRES Plant Nutrition, University of Bonn, Karlrobert-Kreiten-Strasse 13, Bonn, 53115 Germany; 4 Institute for Geodesy and Geoinformation, University of Bonn, Nussallee 17, Bonn, 53115 Germany; 5 Institute for Analysis and Numerics, University of Münster, Einsteinstr. 62, Münster, 48149 Germany

**Keywords:** Tap roots, Shape variation, Energy minimization, 3D point clouds

## Abstract

**Background:**

The efficient and robust statistical analysis of the shape of plant organs of different cultivars is an important investigation issue in plant breeding and enables a robust cultivar description within the breeding progress. Laserscanning is a highly accurate and high resolution technique to acquire the 3D shape of plant surfaces. The computation of a shape based principal component analysis (PCA) built on concepts from continuum mechanics has proven to be an effective tool for a qualitative and quantitative shape examination.

**Results:**

The shape based PCA was used for a statistical analysis of 140 sugar beet roots of different cultivars. The calculation of the mean sugar beet root shape and the description of the main variations was possible. Furthermore, unknown and individual tap roots could be attributed to their cultivar by means of a robust classification tool based on the PCA results.

**Conclusion:**

The method demonstrates that it is possible to identify principal modes of root shape variations automatically and to quantify associated variances out of laserscanned 3D sugar beet tap root models. The introduced approach is not limited to the 3D shape description by laser scanning. A transfer to 3D MRI or radar data is also conceivable.

## Background

In breeding and precision agriculture there is a need for a precise description of the 3D architecture of a crop, a plant organ or a harvested product, in a fast and reproducible way [1].

Potential applications involve automated selection procedures in plant breeding of phenotypes with the desirable features and traits (like grain or root shape), assessment of crop development during the growth period, enabling an optimised crop management and sorting out special forms or non-crop contaminants (stones, weed seeds) of harvested products. An additional application is the adaption of harvester settings, based on archetypal 3D-geometries of harvested cultivars to avoid mechanical damage and by this quality reduction.

Yield formation is the combined result of genetic characteristics and environmental effects. In sugar beets the annual increase in sugar yield accounts 1.5% [2]. This is mainly due to an increase in the root yield, while the sugar content of varieties remained stable [3]. One of the main targets of sugar beet breeding is the root development, including relevant quality parameters such as sugar content, non-sugar compounds or mark-content. It can be observed that the dynamic of storage root development in sugar beet has no specific growth stages. There is no phase of maturation, and therefore, yield development and potential can be estimated depending on the duration of the growing period [3]. Unfortunately, in sugar beet development of leaf biomass is not correlated to storage root biomass or yield formation. However, the shape of the sugar beet tap root plays a significant role for the entire processing chain.

Besides effects of varieties, it is well known that the shape of tap roots mainly depends on the type and status of soil, management and environmental conditions [4, 5]. A strong correlation exists between basic root shape parameters like area, length, or radial variation and sugar yield and quality. Additionally, size and shape is characteristic for cultivars. Factors such as formation of branches or soil-adhesion are relevant breeding traits. On-field devices are proposed to measure and monitor basic parameters of tap roots [6] with varying degrees of success. Shape models are used for the classification of crops and weeds [7]. Image processing methodology already plays a significant role for the observation of plant growth [8]. Computer vision is also used for the analysis of plant root shapes. However, currently these methods are mostly based on 2D imaging modalities [9], whereas the most precise description would result from a robust statistical analysis of the true 3D description of tap roots. First results in imaging the 3D shape of sugar beet have been used to extract scalar parameters of the tap root [10] such as height, width, volume and surface area. By imaging over time the development and growth can be observed. The resulting crop growth model can be improved by a true 4D description of the crop plants [11]. Analysis of 3D point clouds with recent mathematical models or machine learning approaches further improve the efficiency and biological interpretability of plant sensor data [12]. This helps to assess the genotype-phenotype-environment interactions and to dissect important traits [13] [14].

Agricultural crops have to cope with adverse environments but still have to maintain productivity at the highest possible level. Here, breeding has to identify and make use of the best combinations of traits [15, 16]. These traits are mostly quantitative and inherited in a complex manner [13]. Although morphological changes are downstream effects of altered gene expression, metabolic adaptations and environmental changes, their precise, unequivocal, and unbiased identification is of utmost importance in a breeding process to assess Genotype × Environment × Phenotype (*G* ×*E*×*P*) interactions [17]. So far, breeding involves classification of desirable traits (usually around 20) by using empirical scales, which can be assessed mostly by visual assessment in traditional breeding schemes [15]. Considering that there are easily 10,000 individuals or more that have to be classified within a very short time span, the common method is prone to human bias (tiredness, adverse and rapidly changing light conditions). The same holds true for the harvested produce where shapes e. g. of tuber crops have to be assessed. Unequivocal identification of shapes or outlines is of high importance as well for purposes of precision farming where there is a need for an on-the-flight identification of shapes and their assessment, e. g. for the differentiation between crop and weed plants, diseased and healthy plants or plant organs or species identification (see e. g. [18]). Further refinement of phenotyping and the increase of traits that can be simultaneously observed calls for objective and automated ways of trait identification. There have been various attempts so far to describe leaf or tap root shapes with elliptic Fourier analysis [17–19]. The challenge is now to analyze true 3D shape variations into main components, while most methods so far rely on 2D image analysis [17, 20].

Knowledge of the statistical variation of root shapes has various applications. In plant breeding such information can be used to select tendencies with favorable traits, such as an even shape, large crop size, or an optimal form for the use of crop harvesters and to minimize breaking root tips, soil tare and mechanical damages. In crop management the statistical data may help to detect deviations from the expected plant development, which can be used in a feedback mechanism to adjust growth control parameters, such as fertilizer or pesticides.

In this study we apply a computer vision tool for statistical analysis that is purely shape based. Statistical models of shape have been used widely in computer vision and graphics [21]. In a 2D setting, PCA-based models such as Active Shape [22] or Appearance Models [23] provide a parametric representation of shape that can be used for segmentation, tracking and recognition. In a 3D setting, they are typically used for fitting to noisy or ambiguous data or for 3D reconstruction via analysis-by-synthesis. Essentially, the statistical model provides a constraint that significantly reduces the parameter space for many shape processing problems.

It is a wide spread assumption that the input data is collected from a shape space that is considered as a Riemannian manifold. The classical treatment of shape space is due to Kendall [24], in which sets of landmarks are considered points on a shape manifold in which the effects of scale, rotation and translation are factored out. The tangent plane to Kendall’s shape space enables linear principle component analysis (PCA) in which Euclidean distance approximates Procrustes distance. Srivastava et al. [25] propose a representation for analysing shapes of curves under an elastic metric. Killian et al. [26] model the space of triangulated shapes. Statistical analysis can be performed on shape spaces in a manner that respects the Riemannian geometry of the manifold. This requires Riemannian notions of concepts such as distance, mean value and covariance [27]. Based on these quantities Fletcher et al. [28] transferred the concept of PCA to manifolds by considering a principal geodesic analysis (PGA). The idea of PGA was used by Tournier et al. [29] to build a statistical skeleton model and by Heeren et al. [30] to perform a statistical analysis in the space of triangle meshes. However, all these approaches assume the underlying shape space to be a Riemannian manifold. In particular, the dissimilarity of two shapes is quantified by the length of an optimal, connecting curve. In contrast, we here consider a purely elastic model that measures shape dissimilarity by the amount of elastic deformation energy [31]—for details on the physical and geometrical differences we refer to [32]. Using the physical model of 3D elasticity Rumpf and Wirth compute shape averages [33] and describe a covariance analysis [34, 35] of shapes represented as boundary contours of elastic objects. Here the elastic average is defined as the shape that minimizes the sum of elastic deformation energies to all the given input shapes. With an average at hand a classical PCA is then applied to the displacements with respect to the input shapes. This elastic shape analysis was performed on the space of triangle meshes in [36] and will be applied in the present work to the shape space formed by sugar beet tap roots.

In our experiment the acquisition of input shapes is realized based on 3D laser scanning—a method to recover 3D point clouds from objects. This method is well established in the agricultural context and has been used on plants for 3D modelling of the canopy of tomato plants [37], for in-field scanning of pear-trees [38] and imaging physiological responses of leaves [39]. More detailed scans for organ specific parameterization were also possible when using close-up laserscanning. This enables e.g. identification of single organs [10, 12, 40] or tracking of growth on organ level [41], and it has shown to be very accurate [42]. Close-up scanning enables high resolution and high accuracy imaging with point to point distances below a millimeter [43]. This enables highly accurate 3D surface models of various plant types.

**Our contribution.** In this work, we demonstrate the extraction and mathematical description of characteristic shape features in the tap roots of different cultivars of sugar beets. To this end, a huge data set consisting of detailed 3D descriptions of beet root samples is reduced to a small number of important parameters without loosing relevant biological information. Our main contributions are twofold. First, we perform a shape based analysis of laser scanned sugar beet tap roots. In particular, one can compute a robust and reliable shape mean as well as principal modes of shape variation on large ensembles of sugar beets with a substantial variability in the shape geometry. In particular, the statistical tool is not based on predefined quantitative properties (such as length or volume), and it is invariant under rigid body motions. Second, we propose an automatic classification tool based on results from the statistical analysis. The number of tap roots that were classified correctly without having been in the training data set is significantly higher than random classification. This indicates that a cultivar based assignment of a tap root to the corresponding cultivar is possible by only using geometrical shape parameters. The introduced approach is not limited to the 3D shape description by laser scanning and can be generalized for any other 3D measuring device with high precision as structure-from-motion approaches [44] or volume-carving methods [45].

## Results

We introduce a method for the statistical analysis of large ensembles of 3D tap roots as well as a classification tool based on information gained from the statistical analysis. To prove the benefits of our approach, four cultivars with 35 sugar beet tap roots each were measured with a laser scanner. After several pre-processing steps each beet is finally represented as a characteristic function.^1^ In order to validate our classification tool, we randomly removed 5 beets of each cultivar from the training data set. Afterwards, we computed a *shape average* as the minimizer of an elastic matching functional for each of the four training data sets separately. In the same physical setup, a principal component analysis has been applied to the displacements between average and input shapes. This way, we obtained principal modes of main variation within all four training data sets. Again, each of these modes is represented by a displacement of the shape average.

**Fig. 1:**
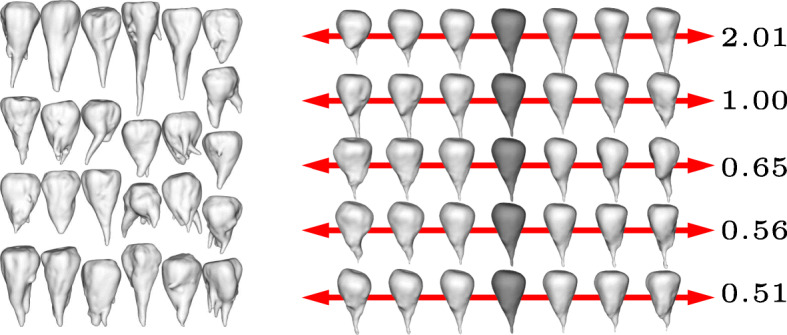
Input beets of sort Berenika (left) and first five modes of main variation (right) with average beet in dark grey and corresponding variances *λ*_1_,…,*λ*_5_ (multiplied by 100)

**Fig. 2:**
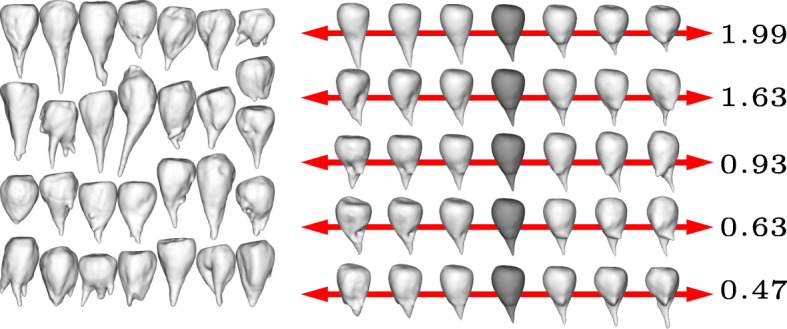
Input beets of sort Cesira (left) and first five modes of main variation (right) with average beet in dark grey and corresponding variances *λ*_1_,…,*λ*_5_ (multiplied by 100)

**Fig. 3:**
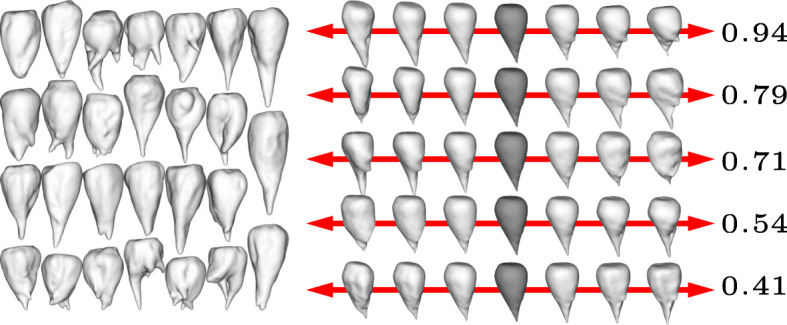
Input beets of sort Mauricia (left) and first five modes of main variation (right) with average beet in dark grey and corresponding variances *λ*_1_,…,*λ*_5_ (multiplied by 100)

**Fig. 4:**
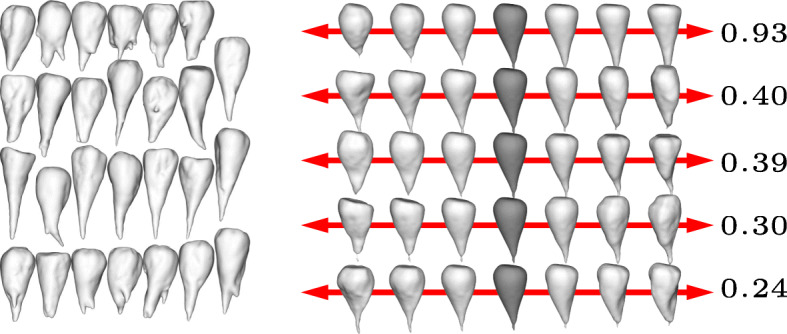
Input beets of sort Pauletta (left) and first five modes of main variation (right) with average beet in dark grey and corresponding variances *λ*_1_,…,*λ*_5_ (multiplied by 100)

The results are shown in Figs. 1 to 4 for the four different cultivars. In each figure we show the input shapes on the left and the five main shape variations on the right, where the middle shape (dark gray) represents the average beet shape. To better illustrate the directions of main variations, the displacements are displayed with different positive and negative magnitudes. Note that all input beets were scaled to have uniform volume, hence the previously most dominant mode of uniform scaling is no longer relevant. However, the relative order within the variances has not been affected substantially by the rescaling. In detail, if $\left (\tilde \lambda _{k}\right)_{k\geq 0}$ describe the variances (in decreasing order) before the uniform rescaling, where $\tilde \lambda _{0}$ is the dominant eigenvalue that represents the impact of uniform scaling, and (*λ*_*k*_)_*k*≥1_ the variances after rescaling, we observe $\lambda _{k-1}/\lambda _{k} \approx \tilde \lambda _{k-1} / \tilde \lambda _{k}$ for *k*>1.

For the variation in cultivar *Berenika* (see Fig. 1) the first mode can be described as a tendency to a multiple or at least double apex (2.01), the second variation as a tendency to a long apex or a more dull one (1.00). For the cultivar *Cesira* (see Fig. 2) the first variation (1.99) is according to the second mode of variation of *Berenika*, the tendency from a long apex to a dull one. The second variation is similar to the first one of *Berenika* (1.63). The main variation for the cultivar *Mauricia* (see Fig. 3) is similar to the long apex or a dull one (0.94), the second variation is the affinity to a multiple or clear and pointy apex (0.79). The first two main variations of *Pauletta* (see Fig. 4) can be described as the affinity to a pointy or dull apex (0.93) and the tendency from a clear and pointy apex to a second apex at the side (0.40). Note that our model is able to capture the tendency of some cultivars to develop multiple apices, although this introduces a strong non-convexity in the modes. This is for example visible in the first two modes of *Berenika* (see Fig. 1) and the third mode of *Mauricia* (see Fig. 3), which represent an initial growth of a second apex from the root body. Even more strikingly, *all* depicted modes of *Cesira* (see Fig. 2) can be associated with the development of apices which is obviously a characteristic feature of the corresponding training data.

In a second step, the principal modes were used to derive a classification tool. Based on the classical *Mahalanobis distance*, we first define a distance measure for each cultivar. Then a given beet is classified to the cultivar that induces the lowest distance. For example, when computing the distance of that given beet to cultivar *Pauletta*, we effectively compute the distance to the average shape of the training data set of *Pauletta*. In particular, the distance measure penalizes deviations in direction of dominant principal modes of this data set less, as these directions are supposed to be characteristic for cultivar *Pauletta*.

**Fig. 5:**
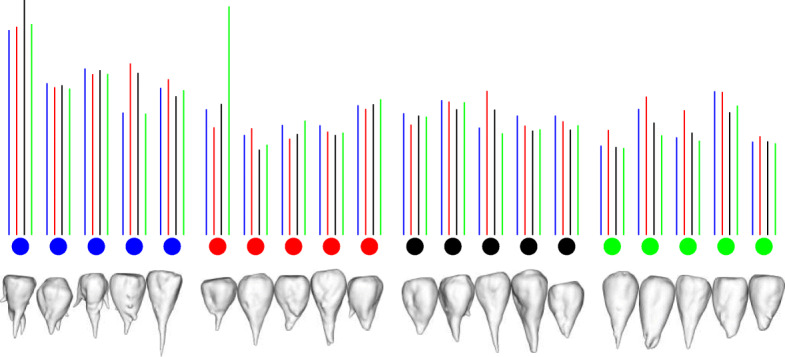
Five beets of sort Berenika (blue), Cesira (red), Mauricia (black) and Pauletta (green) to be classified. Vertical color bars upon each shape are proportional to the distance to the four different cultivars. Overall 55% beets were classified correctly (whereas 25% is random)

**Fig. 6:**
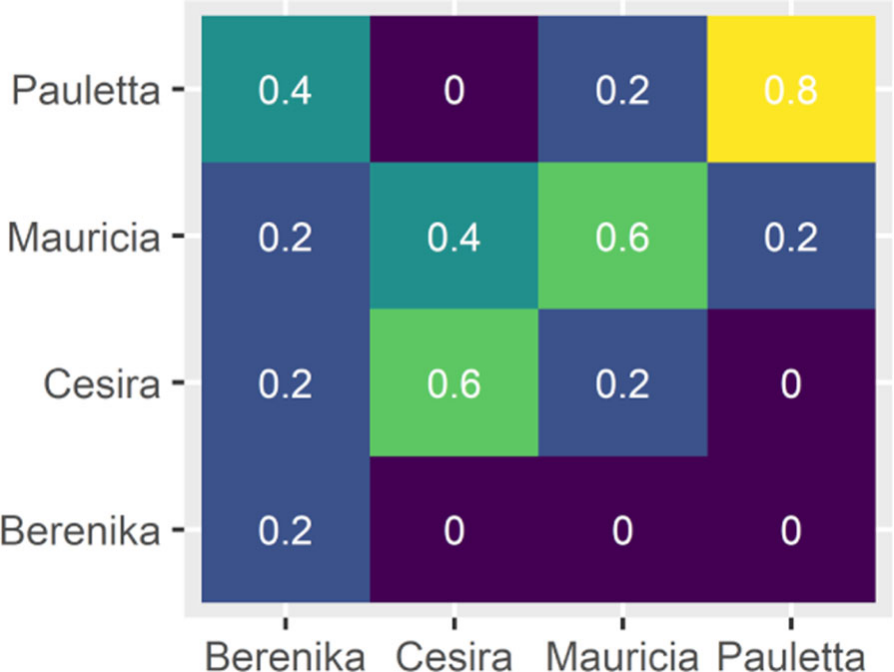
Visualization of results shown in Fig. 5 by means of a confusion matrix to demonstrate differences in classification success for the four different cultivars

Classification results are depicted in Figs. 5 and 6, respectively. In detail, we show for every beet to be classified the (extended) Mahalanobis distance to each of the four cultivars as vertical color bars whose height is proportional to the distance (cf. Fig. 5). For example, the height of the blue bar always represents the distance to the *Berenika* training set. The validation of the classification reveals a significantly better success (i.e. 55%) in comparison to random classification (with a *p*-value of 0.0045). Note that we considered only the first 13 principal modes for each cultivar to design the distance measures (details will be explained in the “Methods” section). If we ignore the principal modes and simply compute the distance to the average we still obtain a correct overall classification rate of 40%. However, we observe huge differences in classification success when distinguishing between cultivars (cf. Fig. 6). In detail, a beet from cultivar *Pauletta* was classified correctly in 80% of the trials, whereas *Berenika* classification success was more or less random (i.e. 20%).

**Simplified PCA approach.** For comparison reasons we also computed a Euclidean principal component analysis on five characteristic parameters extracted directly from the 3D point cloud. In detail, these five different parameters are root length, width, surface and volume (cf. [10]) as well as root complexity which is defined as the quotient between root surface and volume.

**Fig. 7:**
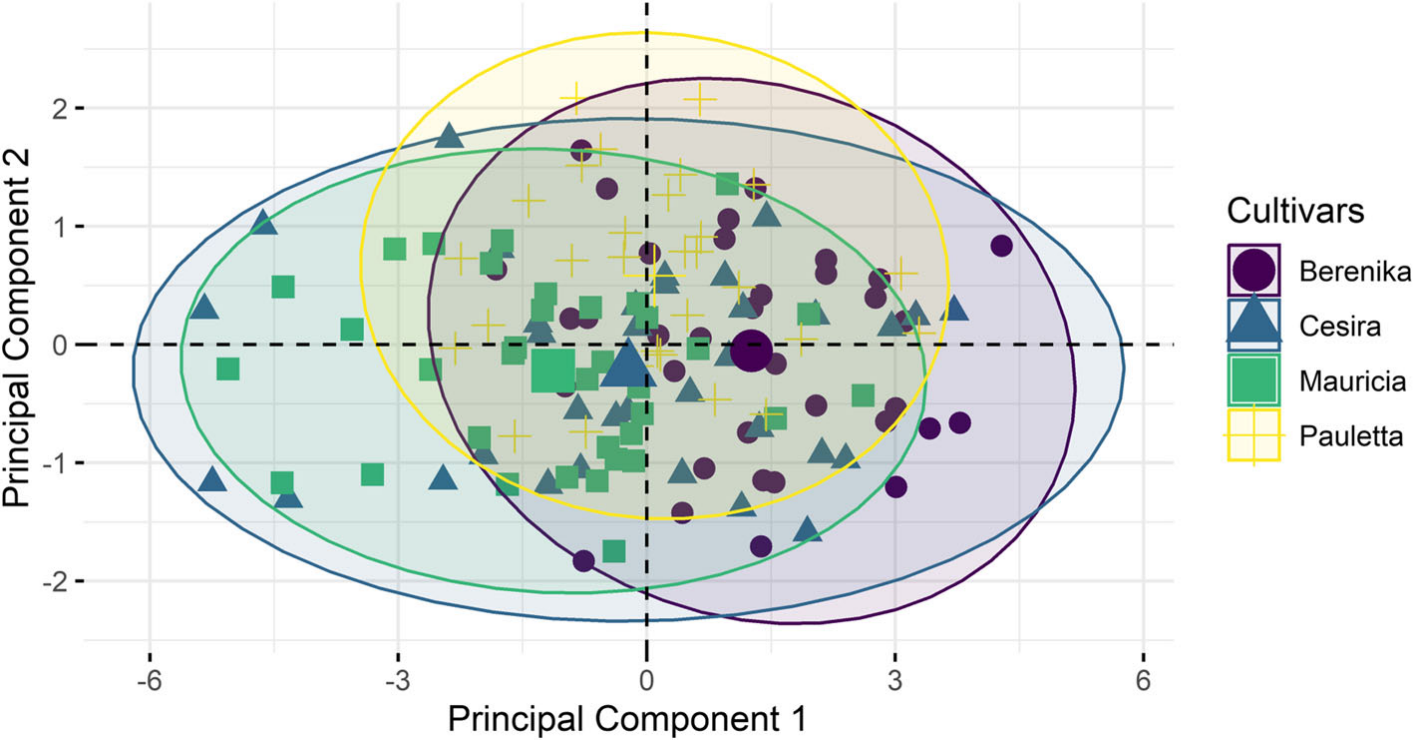
A principal component analysis of measured root traits based on five measured traits for comparison. By using these parameters a differentiation between the cultivars is hard to achieve

Figure 7 shows the distribution of the different cultivars using the first two principal components of the PCA. Obviously, distinguishing different cultivars or even a reliable classification is hard, if not impossible.

## Discussion

The aim of our study was to perform a statistical shape analysis and to develop a method which allows to distinguish between different cultivars in a non-parametric way. Figs. 1 to 4 show how our method identifies the main components of shape variation. These are mostly in line with traits for sugar beet shapes like size, maximum length, maximum width, average radius, radial variation, circularity, the ratio width to length, and further shape factors like surface roughness or furrow formation. Moreover, properties that seem to be characteristic for a particular training data set can actually be extracted in form of dominant variations. For example, the training data set of *Pauletta* (cf. Fig. 4, left) suggests that this cultivar tends to have a rather slim and longish body that varies predominantly in length whereas branching of the main tap root is usually not preferred. On the other hand, the clearly separated first principal mode (*λ*_1_>2*λ*_2_) captures exactly this variation with respect to length and none of the first five modes represents a clear branching (compared to the other cultivars in Figs. 1 to 3). Besides the qualitative modes of variation, our method allows a robust quantification of the associated variance of the main components of 3D shape variation. A next step would be to compare the results also at different environments or sets of stress conditions which enables quantitative statements about the influence of these parameters on the plant’s development. The present outcomes suggest that it will become possible to dissect environmental from genotypic effects on the phenotype and thus allow a true G ×*E*×P interaction analysis [13]. Furthermore, it is envisaged that the proposed method is adopted to other plant organs and species and likewise will be useful for approaches in precision farming.

The results of Fig. 5 have shown a classification accuracy of about 55% which is significantly better than a random assignment. However, the robustness of the classification can still be improved. In particular, the dependence on the number *M*_*j*_ of considered principal modes in the definition of the distance measure has to be further investigated. Furthermore, the striking differences of classification success between different cultivars (cf. Fig. 6) has to be explored and explained in detail. We believe that increasing the number of samples in the training data sets might improve the robustness as well as the classification success of individual cultivars.

Currently, the resolution of the shapes is limited by the spatial resolution of the characteristic functions which are defined on a 129^3^ voxel grid. The choice of spatial resolution is a trade-off between a desired level of details and limitation of computation time. However, the sensor provides a resolution of about 50 microns, hence it is desirable to be able to use the full resolution in the numerical simulations as well. To this end, future work will focus on the reduction of the algorithm’s complexity on the one hand, e.g. by using linearized elasticity and to use more sophisticated algorithmic features on the other hand, such as adaptive grids and advanced parallel computing techniques. An altenative approach is applying the method proposed in [36] to the original triangle meshes constructed directly from the laser scans. This *shell PCA* performs the same statistical shape analysis but with a different physical model (in detail, shapes are treated as hollow objects and one studies elastic deformations of the surface only). Moreover, it might be interesting to investigate the potential application of machine learning approaches to tackle the classification problem. Finally, we aim at the replacement of a manually moved scanner by a device that is able to perform a high number of scans automatically. This replacement is in particular necessary since we aim at increasing the number of samples in the training data sets.

## Conclusions

We applied an established computer vision algorithm to perform a statistical shape analysis on 3D laser scanned tap roots. The method is applied to sugar beets, where the mean root shape and the main variations within a group of sugar beets of the same growth period have been computed. Our investigations can be considered as a case study for the statistical analysis of storage roots without predefined classification criteria. The method indeed demonstrates that it is possible to identify principal modes of shape variation automatically and to quantify the associated variances. In particular, the resulting dominant modes of variations can be used to cluster scanned tap roots into categories which forms the basis for linking growth and environmental conditions. Furthermore, this statistical shape analysis can be used in combination with other invasive or non-invasive sensors that access the 3D shape and can be applied to other plant organs and species as well. Since non-invasive sensors such as MRI or radar imaging are usually affected by significant noise the statistical analysis of large ensembles of laser scanned root shapes could help to increase the accuracy of the classification of noisy input data.

## Methods

**Plant material.** Sugar beets were grown during summer 2012 in a central experiment of CROP.SENSe.net. The objectives of the central experiment were the provision of ground truth data for the interpretation of sensor measurements. The soil was a silty loam soil (WRB: Haplic Luvisol) at the Klein Altendorf experimental research station (6^∘^59’N, 50^∘^37’E) near Bonn, Germany. The mean annual temperature is 9.6 ^∘^C with an average annual precipitation of 625 mm. The cultivars *Pauletta*, *Berenika*, *Mauricia* and *Cesira*—obtained commercially from KWS Saat^2^—were chosen because of their differing phenology. At the end of the growing season 35 randomly chosen beets of each variety were sampled, i.e. 140 beets in total. Rows for sampling were 2 m ×3 m within a plot of 50 m^2^ each. Seed density was 90,000 per hectare at a row distance of 0.2 m (plant to plant distance).

**Fig. 8:**
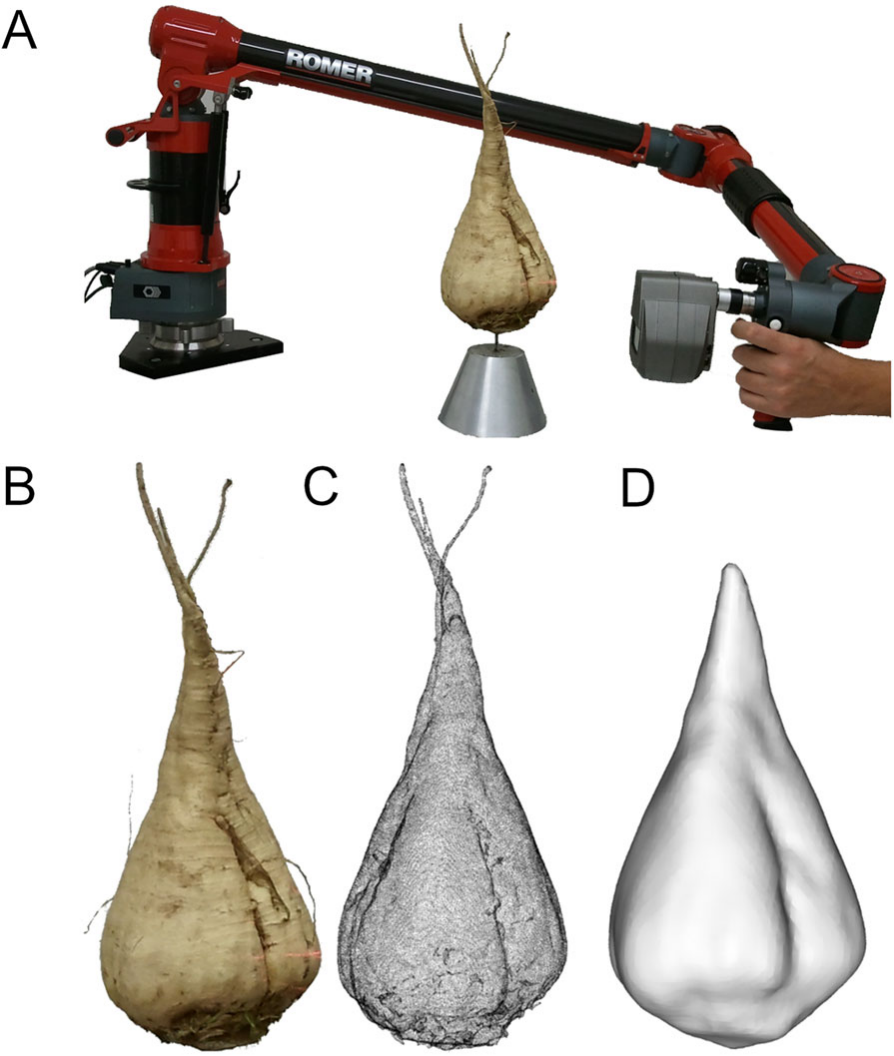
The laserscanner-measuring arm combination with its seven degrees of freedom enables the 3D imaging of the complete sugar beet tap root. It provides a point accuracy of 45*μm* and a point resolution of 17*μm***a**. An RGB image of a sugar beet **b** is shown together with its laserscanned point cloud with a shaded visualization with 3 million points **c** and the down-sampled and smoothed wireframe representation **d**

**Data acquisition.** To acquire the 3D shape of the sugar beet tap roots a close-up laser scanner coupled to an articulated measuring arm device [10, 40] was used (see Fig. 8). This is a well evaluated combination for 3D plant imaging [41, 42]. Hardware details such as resolution and accuracy are given in Table 1. With its seven degrees of freedom it is possible to image the plant from various viewpoints to get an occlusion-free 3D model. The output of the laser scanner is a point cloud with XYZ coordinates with more than 3 million points that is later parsed to an automatic triangulation algorithm.

**Table 1:** Hardware details for the measuring setup

3D scanning hardware	
Manufacturer supporter	Hexagon Metrology Ltd. UK
Model supporter	Romer Infinite 2.0
Manufacturer scanner	Perceptron Inc. Plymouth, MI, USA
Model scanner	Perceptron Scan Works V5
Resolution	17*μm*
Accuracy	45*μm*
Measureable Volume	spherical (1.4m radius)
used Wavelength	660nm
main application field	quality management

**Fig. 9:**
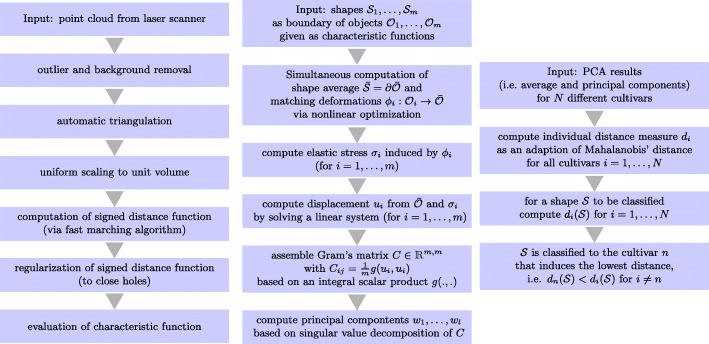
Workflow of our method for data pre-processing (left), statistical analysis (middle) and classification (right)

**Preprocessing (cf. Fig. ****9****, left column).** Before being fed to our statistical method we made use of the commercial 3D-CAD-Software *Geomagic Studio 12* to apply basic preprocessing routines. First, as the sensor is moved manually (see Fig. 8), visible parts from the mounting device and the measuring table had to be removed. Second, the integrated outlier removal function was used as well as a grid-based reduction of the point density (to an average distance of 0.5 mm) to enable a smooth surface generation. Subsequently, the automatic point cloud triangulation was performed to approximate the surface boundary ${{\mathcal {S}}}$ of the solid root shape ${{\mathcal {O}}}$, i.e. ${{\mathcal {S}}} = \partial {{\mathcal {O}}}$. We then applied a uniform rescaling such that all shapes have the same (inscribed) volume.^3^

The proposed statistical method requires the representation of each root shape via a characteristic function $\chi _{{{\mathcal {O}}}}$ on the computational domain *Ω*=[0,1]^3^, i.e. we have $\chi _{{{\mathcal {O}}}}(x) = 1$ if $x \in {{\mathcal {O}}}$ and $\chi _{{{\mathcal {O}}}}(x) = 0$ else. Then, the sugar beet surface ${{\mathcal {S}}}$ is implicitly represented as the interface between these two regions. To obtain such a characteristic function, we first compute the signed distance function $d:\Omega \to {{\mathbbm {R}}}$ of ${{\mathcal {S}}}$, see [46]. In detail, |*d*(*x*)| represents the shortest distance from *x*∈*Ω* to ${{\mathcal {S}}}$, where *d*(*x*)>0 if *x* is in the interior of ${{\mathcal {O}}}$ and *d*(*x*)<0 if $x \notin \bar {{\mathcal {O}}}$. To this end, *Ω*=[0,1]^3^ is discretized by a regular grid *Ω*_*h*_ with 129^3^ nodes (129 equally spaced vertices in each spatial direction of the unit cube). Mathematically, we aim at solving the nonlinar partial differential equation |∇*d*(*x*)|=1 for all *x*∈*Ω*_*h*_ with the boundary condition *d*(*x*)=0 if $x \in {{\mathcal {S}}}$, the so-called *Eikonal equation*. To this end, we first evaluate the signed distance *d*(*x*) on the grid nodes *x*∈*Ω*_*h*_ closest to the triangles of ${{\mathcal {S}}}$. Afterwards, we compute its values far from the triangular surface via information propagation by a fast marching algorithm [47] to obtain the solution $d\colon \Omega _{h} \to {{\mathbbm {R}}}$ of the PDE. Since the triangulated surface might have been non-closed due to local scan deficiencies or self-occlusion, the signed distance function *d* may have an incorrect sign at a number of nodes. As a remedy, we regularize *d* by seeking a minimizer *d*_new_ of the energy

$$\int_{\Omega}\left(|d|_{\epsilon}-|d_{\text{new}}|_{\epsilon}\right)^{2}\,{{\mathrm{d}}} x+\sigma\int_{\Omega}|\nabla d_{\text{new}}|^{2}\,{{\mathrm{d}}} x\,. $$

Here, $|d|_{\epsilon }=\sqrt {|d|^{2}+\epsilon ^{2}}$ with *ε*=10^−4^ denotes a differentiable approximation of the absolute value function and the regularization parameter was set to *σ*=0.01 in our application. The nonlinear optimization is performed using a finite element approach with multilinear basis functions on the regular grid *Ω*_*h*_ and a standard trust region method [48]. Once *d*_new_ is computed we are finally able to define the characteristic function. To this end, we set $\chi _{{{\mathcal {O}}}}(x)=1$ if *d*_new_(*x*)<0 and $\chi _{{{\mathcal {O}}}}(x)=0$ else, which is done for all grid nodes *x*∈*Ω*_*h*_. After data compression, the required memory to store the function $\chi _{{{\mathcal {O}}}}$ is comparable to the one needed to store an explicit triangular representation of the shape ${{\mathcal {S}}}$.

**Statistical analysis model (cf. Fig. ****9****, middle column).** In the following we will generalize the standard statistical analysis of point sets in a (linear) vector space, e. g. ${{\mathbbm {R}}}^{n}$, to the space of shapes **S**, where ${{\mathcal {S}}} \in {{\mathbf {S}}}$ represents the surface of a volumetric object $ {{\mathcal {O}}} = {{\mathcal {O}}}({{\mathcal {S}}}) \subset {{\mathbbm {R}}}^{n} $. To underpin this generalization let us briefly recall the basic concepts of the mean and the principal component analysis (PCA) on vector spaces [49].

The arithmetic mean $\bar x$ of *m* points *x*_1_, *x*_2_, …, *x*_*m*_ in ${{\mathbbm {R}}}^{n}$ is given as $\bar x = \frac 1m (x_{1} + x_{2} + \ldots + x_{m})$. It can also be characterized as the minimizer of the energy $E[x] = {\sum \nolimits }_{i=1}^{m} {{\mathcal {W}}}[x_{i}-x]$, where ${{\mathcal {W}}}[y]=\mu |y|^{2}$ is the elastic energy stored in a spring stretched along a vector *y* and *μ* is the associated stiffness coefficient of the spring. Indeed, the arithmetic mean is the equilibrium position of the central hub of a network with *m* springs, where one end of each spring is attached to one of the input points and the other end is connected to all other springs at a hub so that all springs pull at this hub. If $X = \left [x_{1} - \bar x|\ldots |x_{m} - \bar x\right ]$ is the *n*×*m* (centered) *data matrix*, the covariance matrix is given by $\tfrac 1mXX^{T}$. A PCA now involves a spectral decomposition of this *n*×*n* matrix, where *n* might be very large. Hence we use the fact that the *m*×*m* matrix $C= \tfrac 1m X^{T} X$ has the same (non-trivial) eigenvalues as the covariance matrix, where *m*≪*n*. Note that $C_{ij} = \frac 1m g\left (x_{i}-\bar x,x_{j}-\bar x\right)$ where *g* denotes a suitable scalar product on ${{\mathbbm {R}}}^{n}$ (typically the standard Euclidean inner product $\left (x_{i}-\bar x\right)\cdot \left (x_{j}-\bar x\right)$). If *C*=*QΛQ*^*T*^ is the singular value decomposition of *C* with an orthogonal matrix *Q* and a diagonal matrix *Λ* with diagonal entries *λ*_1_≥*λ*_2_≥…≥*λ*_*m*_, then the eigenvectors of the covariance matrix are obtained via $w_{j} = {\sum \nolimits }_{i=1}^{m} \sqrt {\lambda _{j}^{-1}} q_{j}^{i}\left (x_{i}-\bar x\right)$. These are exactly the principal directions of variations, and the eigenvalues *λ*_*j*_ describe the variance in that direction. Here, $q_{j}^{i}$ denotes the *i*^th^ entry in the *j*^th^ column *Q*. As *g* is supposed to be positive (semi-)definite, we have *λ*_*i*_≥0 for *i*=1,…,*m*. Furthermore, if we assume *x*_1_,…,*x*_*m*_ to be linearly independent we have *λ*_1_≥…≥*λ*_*m*−1_>*λ*_*m*_=0 as we consider centered data, i.e. $x_{1}-\bar x, \ldots, x_{m} - \bar x$ spans a (*m*−1)-dimensional subspace.

Now this general concept is transferred to shape statistics. In the context of tap root shapes ${{\mathcal {S}}}_{i}$ for *i*=1,…,*m*, the corresponding volumetric objects ${{\mathcal {O}}}_{i} = {{\mathcal {O}}}\left ({{\mathcal {S}}}_{i}\right)$ play the role of the points *x*_*i*_, and the vector *x*_*i*_−*x* is replaced by the optimal deformation $\phi _{i}:{{\mathcal {O}}}_{i}\to {{\mathbbm {R}}}^{n}$ of the object ${{\mathcal {O}}}_{i}$ into an object ${{\mathcal {O}}}$. Here, optimal means that the deformation *ϕ*_*i*_ costs the least elastic energy ${{\mathcal {W}}}\left [\psi _{i}, {{\mathcal {O}}}_{i}, {{\mathcal {O}}}\right ] = \int _{{{\mathcal {O}}}_{i}} W\left (D \psi _{i}\right) {{\mathrm {d}}} x$ of all deformations with $\psi _{i}({{\mathcal {O}}}_{i}) = {{\mathcal {O}}}$, where *W*(·) denotes a hyperelastic energy density acting on the local deformation gradient $D \psi _{i}\in {{\mathbbm {R}}}^{n\times n}$. This energy and thus the thereby defined shape mean is invariant under rigid body motions, i. e. it does not change if the position or the orientation of the root shapes is varied. The arithmetic mean of *m* input objects ${{\mathcal {O}}}_{1},\,{{\mathcal {O}}}_{2},\, \ldots, \, {{\mathcal {O}}}_{m}$ is defined as the object $\bar {{\mathcal {O}}}$ which minimizes the energy

$$\begin{array}{*{20}l} E[{{\mathcal{O}}}] = \sum\limits_{i=1}^{m} {{\mathcal{W}}}\left[\phi_{i},{{\mathcal{O}}}_{i},{{\mathcal{O}}}\right]\,. \end{array} $$

**Fig. 10:**
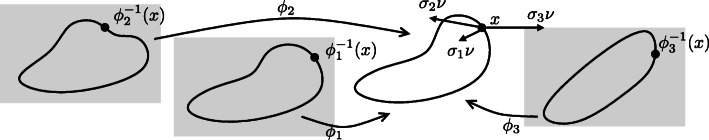
Sketch of the stresses *σ*_*i*_ induced by the deformations *ϕ*_*i*_ on the averaged shape $\bar {{\mathcal {S}}} = \partial \bar {{\mathcal {O}}}$, here for *i*=1,2,3

For details we refer to [33]. Different from the vectors $x_{i}-\bar x$ above, the deformations *ϕ*_*i*_ are strongly nonlinear and cannot be used in a (linear) PCA. Instead, one can replace the deformation *ϕ*_*i*_ by its linear representative, the associated elastic stress (cf. Fig. 10)

$$\sigma_{i} = W'\left(\left(D\left(\phi_{i}^{-1}\right)\right)^{-1}\right) \left(\det D\left(\phi_{i}^{-1}\right)\right) \left(D\left(\phi_{i}^{-1}\right)\right)^{-T} \nu, $$

evaluated on the average shape $\bar {{\mathcal {S}}}$ (the surface of the average object $\bar {{\mathcal {O}}}$) with surface normal vector *ν* [31]. Even more intuitive, one can also replace the nonlinear deformation *ϕ*_*i*_ by the displacement $u_{i}:\bar {{\mathcal {S}}}\to {{\mathbbm {R}}}^{n}$ of each point on the surface $\bar {{\mathcal {S}}}$ of $\bar {{\mathcal {O}}}$ which is observed if the elastic stress *σ*_*i*_ is applied at the surface of the average object. The Hessian of the corresponding elastic energy implies a natural scalar product *g*(·,·) on these displacements *u*_*i*_ [31, 35]. Thus, we define the matrix

$$C = \frac1m \left(g\left(u_{i},u_{j}\right)\right)_{i,j=1,\ldots, m} $$

as a representation of the covariance operator, which is defined by

$${{\mathbf{Cov}}} \, u = \frac1m \sum\limits_{i=1}^{m} g(u_{i}, u) u_{i} $$

and can be regarded as the analogon of the covariance matrix in the vector space case. Again, the whole construction is rigid body motion invariant. Via the same spectral decomposition *C*=*QΛQ*^*T*^ of the *m*×*m* matrix *C* as above, we finally obtain the principal modes of shape variation,

$$w_{j}(x)= \sum\limits_{i=1}^{m} \sqrt{\lambda_{j}^{-1}} q_{j}^{i} u_{i}(x), $$

and associated variances *λ*_*j*_. A comprehensive introduction to this concept can be found in [32, 34]. Let us emphasize that this statistical approach does not make any assumptions on how the different shapes and their variations are configured. It is also not assumed that the original shapes are actually elastic—this is just a mathematical tool to define the dissimilarity between shapes.

**Classification (cf. Fig. ****9****, right column).** Sample beets were available for *N*=4 different cultivars. The training data set of cultivar *j* is represented as a set of displacements $\left (u^{j}_{k}\right)_{k\leq K_{j}}$ from the group average $\bar {{\mathcal {S}}}_{j}$, where *K*_*j*_ denotes the sample size. The aim of classification is to assign an arbitrary shape ${{\mathcal {S}}}$ or a corresponding shape displacement *u*, respectively, to one of these *N* cultivars. In detail, we need a distance measure *d*_*j*_ for each cultivar that quantifies the distance of *u* to the set $\left (u^{j}_{k}\right)_{k\leq K_{j}}$. We say that *u* is classified to cultivar *l* if *d*_*l*_(*u*)≤*d*_*j*_(*u*) for *j*=1,…,*N*.

Our distance measure *d*_*j*_ is an extension of the classical Mahalanobis distance. First, we assume that we have already performed a PCA (as described above) for each cultivar independently. Let $\left (\lambda ^{j}_{k}\right)_{k\leq K_{j}}$ and $\left (w^{j}_{k}\right)_{k\leq K_{j}}$ denote the eigenvalues and eigenvectors computed in the PCA of cultivar *j*, respectively. We define *U*_*j*_ to be the linear span of $\left (u^{j}_{k}\right)_{k\leq K_{j}}$ and $U_{j}^{\perp }$ the orthogonal complement with respect to the metric *g*_*j*_(.,.) associated by the *j*th cultivar. For some truncation value *M*_*j*_≤*K*_*j*_ the classical (squared) Mahalanobis distance is given by

$$ m_{j}^{2}(u) := \sum\limits_{k=1}^{M_{j}} \frac{g_{j}\left(u, w_{k}^{j}\right)^{2}}{\lambda_{k}^{j} }\,. $$

Note that $m_{j}^{2}\left (w^{j}_{k}\right) = \left (\lambda ^{j}_{k}\right)^{-1}$, that means the Mahalanobis distance penalizes deviations in direction of dominant eigenvectors less. On the other hand, we have *m*_*j*_(*u*^⊥^)=0 for each $u^{\perp } \in U_{j}^{\perp }$, which requires some kind of regularization. If *P*_*j*_*u* is a projection of *u* onto *U*_*j*_ we decompose $u = P_{j} u + \left (u\,-\,P_{j} u\right) \in U_{j} \oplus U_{j}^{\perp }$ and finally define an *extended Mahalanobis distance* by

$${d}_{j}^{2}(u) = m_{j}^{2}\left(P_{j} u\right) + \frac1\beta g_{j}\left(u\,-\,P_{j} u, u\,-\,P_{j} u \right) \,. $$

Here *β*>0 is a regularization parameter. In the experiments shown in Fig. 5 we have chosen *β*=10^−5^ and *M*_*j*_=13 for all four cultivars.

**Numerical implementation.** The matching deformations *ϕ*_*i*_ between an input object ${{\mathcal {O}}}_{i}$ and the object ${{\mathcal {O}}}$, both represented by characteristic functions *χ*_*i*_ and *χ*, respectively, are computed using a penalty approach, where one minimizes

$$\int_{{{\mathcal{O}}}_{i}} W(D \phi) {{\mathrm{d}}} x+ \gamma \int_{[0,1]^{3}}\left(\chi_{i}-\chi \circ\phi\right)^{2}\,{{\mathrm{d}}} x $$

(for some penalty parameter *γ*=50) with respect to the deformation *ϕ*. The first term ensures that the deformation with minimum energy is found, and the second term ensures that *ϕ* indeed deforms ${{\mathcal {O}}}_{i}$ into ${{\mathcal {O}}}$. In detail, we made use of the polyconvex energy density $W(D\phi) = \bar W(\|D\phi \|, \det D\phi)$ with

$$\bar W(a,d) = \frac\mu2 a^{2} +\frac\lambda4 d^{2}-\left(\mu+\frac\lambda2\right)\log d-\frac{3\mu}2-\frac\lambda4\,, $$

where the elastic parameters are *μ*=*λ*=1. Consequently, the average shape $\bar {{\mathcal {S}}}$, represented by *χ*, and the deformations *ϕ*_1_,…,*ϕ*_*m*_ are obtained by minimizing

$$\sum\limits_{i=1}^{m} \int_{{{\mathcal{O}}}_{i}} W\left(D \phi_{i}\right) {{\mathrm{d}}} x+\gamma \int_{[0,1]^{3}}\left(\chi_{i}-\chi \circ\phi_{i}\right)^{2}\,{{\mathrm{d}}} x $$

over all *ϕ*_*i*_ and *χ*. For details we again refer to [33]. The functions *χ*_*i*_ and deformations *ϕ*_*i*_ are discretized by 129^3^ nodes (i. e. a function value is assigned to each node of a grid with 129 vertices in each space direction) so that the number of degrees of freedom scales with 129^3^·3·*m*, yielding a high-dimensional problem. The principal component analysis basically involves a singular value decomposition of a symmetric *m*×*m* correlation matrix and the solution of *m* linear systems of equations of size 129^3^ in order to translate the boundary stresses into displacements or shape variations.

## Data Availability

The datasets used and analysed during the current study are available from the corresponding author on reasonable request.

## Abbreviations

*G* ×*E*×*P*: Genotype ×Environment × Phenotype
PCA: principle component analysis
PDE: partial differential equation

## Footnotes

1 A characteristic function assigns 1 = *t**r**u**e* to every data point inside the sugar beet volume and 0 = *f**a**l**s**e* to all other points.

2 www.kws.de

3 The rescaling proved to improve the later classification substantially while it does not qualitatively change the shape analysis. See discussion above.
